# Supplementary material for: Colorectal Cancer in Inflammatory Bowel Diseases: Epidemiology and Prevention: A Review
Source: Cancers (Basel). 2022 Aug 31;14(17):4254. doi: 10.3390/cancers14174254 (PMC9454776; doi:10.3390/cancers14174254)
Supplement: Supplementary file 1 [file cancers-14-04254-s001.zip › cancers-1823554-supplementary.pdf]

Supplementary Table S1. Search strategy of electronic databases

**PubMed search**	(“inflammatory bowel diseases”[MeSH Terms] OR (“inflammatory”[All Fields] AND “bowel”[All Fields] AND “diseases”[All Fields]) OR “inflammatory bowel diseases”[All Fields] OR (“inflammatory”[All Fields] AND “bowel”[All Fields] AND “disease”[All Fields]) OR “inflammatory bowel disease”[All Fields]) AND (“prevention and control”[Subheading] OR (“prevention”[All Fields] AND “control”[All Fields]) OR “prevention and control”[All Fields] OR “prevention”[All Fields]) OR (“inflammatory bowel diseases”[MeSH Terms] OR (“inflammatory”[All Fields] AND “bowel”[All Fields] AND “diseases”[All Fields]) OR “inflammatory bowel diseases”[All Fields]) AND (“epidemiology”[Subheading] OR “epidemiology”[All Fields] OR “epidemiology”[MeSH Terms]) OR (“inflammatory bowel diseases”[MeSH Terms] OR (“inflammatory”[All Fields] AND “bowel”[All Fields] AND “diseases”[All Fields]) OR “inflammatory bowel diseases”[All Fields] OR (“inflammatory”[All Fields] AND “bowel”[All Fields] AND “disease”[All Fields]) OR “inflammatory bowel disease”[All Fields]) AND (“epidemiology”[Subheading] OR “epidemiology”[All Fields] OR “surveillance”[All Fields] OR “epidemiology”[MeSH Terms] OR “surveillance”[All Fields]) OR (“crohn disease”[MeSH Terms] OR (“crohn”[All Fields] AND “disease”[All Fields]) OR “crohn disease”[All Fields]) AND (“epidemiology”[Subheading] OR “epidemiology”[All Fields] OR “epidemiology”[MeSH Terms]) OR (“crohn disease”[MeSH Terms] OR (“crohn”[All Fields] AND “disease”[All Fields]) OR “crohn disease”[All Fields]) AND (“prevention and control”[Subheading] OR (“prevention”[All Fields] AND “control”[All Fields]) OR “prevention and control”[All Fields] OR “prevention”[All Fields]) OR ((“ulcer”[MeSH Terms] OR “ulcer”[All Fields] OR “ulcerative”[All Fields]) AND (“colitis”[MeSH Terms] OR “colitis”[All Fields])) AND (“epidemiology”[Subheading] OR “epidemiology”[All Fields] OR “epidemiology”[MeSH Terms]) OR ((“ulcer”[MeSH Terms] OR “ulcer”[All Fields] OR “ulcerative”[All Fields]) AND (“colitis”[MeSH Terms] OR “colitis”[All Fields])) AND (“prevention and control”[Subheading] OR (“prevention”[All Fields] AND “control”[All Fields]) OR “prevention and control”[All Fields] OR “prevention”[All Fields])
**Embase search**	(‘Inflammatory bowel diseases’/exp OR ‘c’ OR ‘crohn disease’/exp OR ‘ulcerative colitis’) AND (‘epidemiology’/exp OR epidemiology))) AND (‘prevention’/exp OR ‘prevention’)
**Medline search**	(inflammatory bowel diseases AND epidemiology) OR (inflammatory bowel diseases AND prevention)
